# Supplementary material for: Sport Nutrition Knowledge, Attitudes, Sources of Information, and Dietary Habits of Sport-Team Athletes
Source: Nutrients. 2022 Mar 23;14(7):1345. doi: 10.3390/nu14071345 (PMC9002668; doi:10.3390/nu14071345)
Supplement: Supplementary file 1 [file nutrients-14-01345-s001.zip › nutrients-1631122-supplementary.pdf]

Supplementary Material S1: the NUKYA questionnaire.

This test consists of questions on different aspects of sports nutrition. The results will provide us with useful information to establish how we can help you with these issues.

1. 1. Do these foods have a high or low content of complex carbohydrates? For each food choose one of the 3 options (High, Low, Not sure / I don't know)

	High	Low	I am not sure/ I don't know
1.1 Chicken	❑	❑	❑
1.2 Honey	❑	❑	❑
1.3 Beans (pulses)	❑	❑	❑
1.4 Bread	❑	❑	❑
1.5 Jam	❑	❑	❑
1.6 Butter	❑	❑	❑
1.7 Breakfast cereal	❑	❑	❑
1.8 Rice	❑	❑	❑
1.9 Candies	❑	❑	❑

2. Should an athlete who wants to lose weight completely eliminate carbohydrates from his diet?

❑ Yes	❑ No	❑ I am not sure/ I don't know

3. Are carbohydrates stored in the muscle as glycogen?

❑ Yes	❑ No	❑ I am not sure/ I don't know

4. Does the muscle use protein as the main source of energy during exercise?

❑ Yes	❑ No	❑ I am not sure/ I don't know

5. Do these foods have a high or low content of protein? For each food choose one of the 3 options (High, Low, Not sure / I don't know)

	High	Low	I am not sure/ I don't know
5.1 Chicken	❑	❑	❑
5.2 Beans (pulses)	❑	❑	❑
5.3 Fruit	❑	❑	❑
5.4 Margarine / butter	❑	❑	❑
5.5 Breakfast cereals	❑	❑	❑
5.6 Nuts	❑	❑	❑

6. Do fats play an important role in the body?

❑ Yes	❑ No	❑ I am not sure/ I don't know

7. Do saturated and unsaturated fats have the same impact on health?

❑ Yes	❑ No	❑ I am not sure/ I don't know

8. Do these foods have a high or low content of unsaturated fat? For each food choose one of the 3 options (High, Low, Not sure / I don't know)

	High	Low	I am not sure/ I don't know
8.1 Chicken	❑	❑	❑
8.2 Nuts	❑	❑	❑
8.3 Avocado	❑	❑	❑
8.4 Fish and seafood	❑	❑	❑
8.5 Cheeses	❑	❑	❑
8.6 Sausages	❑	❑	❑
8.7 Breakfast cereals	❑	❑	❑
8.8 Lettuce	❑	❑	❑
8.9 Olive oil/ sunflower	❑	❑	❑

9. How many servings of fruits and vegetables is recommended to eat per day? One serving is a piece of fruit or a bowl of salad or vegetables. (Choose an option)

- ❑ 1 or 2 day
- ❑ 3 or 4 day
- ❑ 5 or + day
- ❑ I am not sure/ I don't know

10. Are the following statements true or false?

a. Your athletic performance will decrease if you lose 2% of your body weight (for example, 1.5 kg if you weigh about 75 kg) due to water loss.

❑ True	❑ False	❑ I am not sure/ I don't know

b. To be well hydrated during sports training you have to wait until you are thirsty to drink.

❑ True	❑ False	❑ I am not sure/ I don't know

c. To fully rehydrate after exercise, you need to drink a volume of liquid greater than the volume of water lost during exercise (which we know by weighing yourself before and after training or competition).

❑ True	❑ False	❑ I am not sure/ I don't know

d. Fruit juice is a liquid suitable to drink in the training session and in the middle of the match.

❑ True	❑ False	❑ I am not sure/ I don't know

e. Energy drinks like "Red Bull" are recommended for athletes to ingest during exercise.

❑ True	❑ False	❑ I am not sure/ I don't know

11. What do you think is the most suitable urine color before starting to train? (Choose an option)
- ❑ Clear
- ❑ Pale yellow / (lemon juice)
- ❑ Rich yellow / (apple juice)
- ❑ I am not sure/ I don't know

12. During intense or prolonged exercise, what is the best way to replace the water that is lost in the form of sweat? (Choose an option)
- ❑ Water alone
- ❑ Water and mineral salts
- ❑ Water and carbohydrates
- ❑ Water, carbohydrates and minerals
- ❑ I am not sure/ I don't know

13. The percentage of carbohydrates in an isotonic sports drink should be: (Choose an option)
- ❑ 4-6%
- ❑ 6-8%
- ❑ 8-10%
- ❑ 10-12 %
- ❑ I am not sure/ I don't know

14. What is the optimum time to eat and drink something for kick-starting recovery after exercise or competition? (Choose an option)
- ❑ As soon as possible (during the first 2 hours post exercise)
- ❑ Between the 2nd and 3rd hours post-exercise
- ❑ After the 3rd hour post-exercise
- ❑ When I am hungry
- ❑ I am not sure/ I don't know

15. The most important nutrient(s) to ingest after training is (are): (Choose an option)
- ❑ Water
- ❑ Carbohydrates
- ❑ Protein
- ❑ Fat
- ❑ Water+carbohydrates+protein
- ❑ I am not sure/ I don't know

16. Should the last main meal (breakfast, lunch or dinner) be eaten at least 3-4 hours before a competition / exercise?

❑ Yes	❑ No	❑ I am not sure/ I don't know

17. Can the human body get most of its vitamin D from sunlight exposure?

❑ Yes	❑ No	❑ I am not sure/ I don't know

18. Are vitamins and minerals a good source of energy?

❑ Yes	❑ No	❑ I am not sure/ I don't know

19. Are these foods a good source of iron?

	Yes	No	I am not sure/ I don’t know
19.1 Avocado	❑	❑	❑
19.2 Meat (in general)	❑	❑	❑
19.3 Fish and seafood	❑	❑	❑
19.4 Beans (pulses)	❑	❑	❑
19.5 Spinach	❑	❑	❑
19.6 Nuts	❑	❑	❑
19.7 Bread	❑	❑	❑
19.8 Butter	❑	❑	❑

20. Are these foods a good source of calcium?

	Yes	No	I am not sure/ I don’t know
20.1Fruit	❑	❑	❑
20.2 Meat (in general)	❑	❑	❑
20.3 Almonds	❑	❑	❑
20.4 Milk	❑	❑	❑
20.5 Spinach	❑	❑	❑
20.6 Cheese	❑	❑	❑
20.7 Bread	❑	❑	❑
20.8 Butter	❑	❑	❑
